# Multiple myeloma presenting as spinal cord compression: a case report

**DOI:** 10.1186/1752-1947-4-251

**Published:** 2010-08-06

**Authors:** Chayan Chakraborti, Kristen L Miller

**Affiliations:** 1 Department of Internal Medicine, Tulane University Health Sciences, New Orleans, Louisiana, 70112, US; 2 Department of Internal Medicine, University of Virginia School of Medicine, Charlottesville, Virginia, 22908, US

## Abstract

**Introduction:**

Spinal cord compression is a potentially devastating condition that demands immediate attention. Efforts must be divided between addressing the symptoms of cord compression and identifying the precise etiology of the condition.

**Case presentation:**

A 76-year-old Peruvian man presented to our emergency department for evaluation of the gradual onset of lower extremity weakness over one month, resulting in falls and a two day history of bladder and bowel incontinence. Surprisingly, the etiology of this case of spinal cord compression was found to be multiple myeloma presenting as a solid tumor.

**Conclusion:**

We report a case of a spinal cord mass resulting in symptoms of cord compression that was diagnosed when aspects of our patient's initial magnetic resonance imaging scan did not correlate with disc herniation, which was the diagnosis with the greatest pretest probability.

## Introduction

Spinal masses are prevalent in medicine. These masses most often result from a metastatic primary neoplasm, although many other etiologies are possible. They present most commonly as pain (both local and radicular), weakness, paresthesias, loss of bladder or bowel function or ataxia. These are all signs of spinal cord compression. Early recognition of spinal masses and compression symptoms, in addition to identifying the underlying cause, is crucial as delay in treatment can have devastating consequences.

## Case presentation

A 76-year-old Peruvian man presented to the emergency department for evaluation of one month of gradual onset of lower extremity weakness resulting in falls. He also reported a two day history of bladder and bowel incontinence. A systemic review of our patient was notable for dull but intense chronic back pain. He was no longer ambulatory, had lower extremity numbness and tingling, and had experienced an unspecified amount of weight loss over the last six months. A systemic review of our patient was otherwise unremarkable.

Our patient had emigrated from Peru to the United States seven years prior to this admission and had not been seen by a physician until the current admission. His medical history was significant for iron deficiency anemia, a cholecystectomy (reason unknown), a hernia repair, and a prostatectomy one year prior to his emigration to the United States. The prostatectomy was reported to be for symptomatic benign prostatic hypertrophy.

Physical examination of our patient revealed the absence of bilateral lower extremity reflexes, lower extremity weakness (one out of five), upper extremity weakness (three out of five), mild saddle anesthesia and tenderness along his spine. Sensation to pain and temperature, as well as proprioception, was absent in his lower extremities. Aside from mild paresthesia, sensation in his upper extremities was intact. Other findings on physical examination were unremarkable.

Other than his hemoglobin of 12.1 g/dL (normal range is 13.5 to 17.5 g/dL) and a mildly elevated BUN-to-creatinine ratio at 28 mg/dL (normal range is 7 to 18 mg/dL) to 1.2 mg/dL (normal range is 0.6 to 1.2 mg/dL), our patient's laboratory values were within normal limits. Results for corrected serum calcium and coagulation studies were normal. His total protein level was 5.8 g/dL (normal range = 6 to 8 g/dL), and his albumin level was 3.2 g/dL (normal range is 3.5 to 5 g/dL).

His alkaline phosphatase was 142 U/L (normal range is 40 to 125 U/L). Radiographic studies on admission included a normal chest radiograph and a normal non-contrast computed tomography (CT) scan of his brain. Magnetic resonance imaging (MRI) with gadolinium of his lumbar spine showed both left-sided L2-3 and right-sided L4-5 degenerative disc disease with protrusion into the neural foramen and multiple foci of abnormal bone marrow signal enhancement. A subsequent MRI of his cervical spine showed a large mass at the cervicothoracic junction extending from C7 to T1, bony destruction of three vertebral bodies and epidural extension causing severe spinal cord compression and cord edema. CT scans of his neck, thorax and abdomen did not identify a primary neoplasm, but did note the cervical mass with nodular hemorrhagic areas and numerous well-defined lytic lesions of his axial and appendicular skeleton and ribs.

Common tumor markers (CEA, CA 19-9, and PSA) were found to be normal. Serum protein electrophoresis demonstrated hypoproteinemia with hypoalbuminemia and borderline low gamma globulins. Urine protein electrophoresis showed a band of restricted mobility in the globulin region. Immunofixation revealed monoclonal light chains.

On examination, a pathological specimen obtained through CT-guided biopsy revealed soft tissue necrosis and sheets of mature plasma cells. The cells stained positive for CD138 and CD79a, thus confirming plasma cell lineage. Bone marrow aspirate displayed a focally hypercellular bone marrow with mild trilinear hyperplasia, mild to moderate plasmacytosis (5% to 20%) and iron changes consistent with a state of chronic disease. These results, together with protein electrophoresis and radiographic images, confirmed the diagnosis of multiple myeloma.

## Discussion

This case presented a challenge in that our patient's initial presentation had a preponderance of lower extremity symptoms compared to upper extremity symptoms. Thus, his pretest probability was highest for conditions affecting the lumbar spine, such as cauda equine syndrome from disc herniation or metastatic disease. The initial MRI of his lumbar spine in fact confirmed disc herniation with protrusion, but the abnormal bone marrow signal enhancement came as a surprise. We investigated the extent of his bone marrow abnormalities through further MRI imaging. Cervical imaging revealed the etiology, despite the mildness of the upper extremity symptoms.

The mass may have represented a benign tumor, such as osteoblastoma, giant cell tumor, aneurismal bone cyst, hemangioma, eosinophilic granuloma or angiolipoma. It may have also represented a primary malignancy such as (in decreasing order of prevalence), solitary plasmacytoma, chordoma, chondrosarcoma, lymphoma, Ewing's sarcoma, osteosarcoma, fibrosarcoma, malignant giant cell tumor, or angiosarcoma [1]. MRI findings provided evidence against many of these diagnoses, as well as against primary intramedullary central nervous system neoplasms, such as ependymoma or astrocytoma, which are more common in children than in adults [2].

Our patient's travel history brings into consideration tuberculosis, particularly as an infection of the vertebral body (Pott's disease, tuberculous spondylitis, or tuberculoma), which most commonly manifests in adults [3,4]. The absence of tuberculosis in other locations does not exclude the diagnosis. Tuberculomas can have associated collapsed vertebrae and present with numbness, paraplegia and bladder disturbances similar to this presentation. However, but this would be an extremely atypical presentation of tuberculoma [4].

Other granulomatous diseases, such as sarcoidosis, were also considered as neurosarcoid lesions can resemble a tumor. Spinal cord involvement can occur as part of systemic sarcoidosis, either as the first manifestation or later in the course of the disease as in fewer than 1% of reported cases [5]. The presenting symptoms can be paraparesis, sensory changes or cauda equina syndrome, with the cervical spine being the spinal cord segment most frequently involved [5].

With the numerous lytic lesions throughout the skeleton, multiple myeloma with plasmacytoma formation was the most likely systemic illness. However, given our patient's age, lack of primary care, weight loss, and prostatectomy, metastatic prostate cancer initially remained at the forefront of our differential diagnosis, followed by plasmacytoma.

Primary bone neoplasms account for fewer than 10% of all cases of bone tumors, with metastatic lesions far more widespread in the adult population [1]. Bone metastases, including those to the spine, are a frequent complication of cancer (approximately 5%), occurring most commonly in prostate cancer (up to 70% of patients) and 15% to 30% of patients with cancer of the lung, colon, stomach, bladder, rectum, thyroid and kidney [6]. Both osteolytic and osteoblastic metastases can cause pathologic fractures and subsequent spinal cord compression [6].

Multiple myeloma represents 1% of all cancers diagnosed in the United States and 10% of all hematologic cancers. The annual incidence is 3 to 4 cases per 100,000 population, with the median age of diagnosis in the mid-sixties [7-9]. Multiple myeloma is a condition of malignant plasma cell proliferation derived from a single B-cell lineage [7,8]. These cells produce monoclonal immunoglobulins, most commonly either immunoglobulin G (IgG) or immunoglobulin A (IgA) [10]. Making the diagnosis includes demonstrating these M-proteins in either serum or urine, proving the presence of more than 10% of these malignant plasma cells in the bone marrow and observing the clinical manifestations of the disease in our patient [7,8,10].

As a gammopathy, multiple myeloma generally presents with recurrent infections secondary to humoral immune deficiencies, or with bone pain as a result of osteolytic lesions. Other common presentations include systemic sequelae such as renal insufficiency due to light chain deposition, anemia, fatigue, and hypercalcemia [7-10]. Up to 30% of patients are diagnosed incidentally while being evaluated for unrelated problems, while another third are diagnosed following a fracture [7]. The incidence of bone pain from osteolytic lesions ranges from 58% [8] to 66% [7] of patients with myeloma. Spinal cord compression following vertebral compression fractures or vertebral plasmacytomas comprises 5% of the presentations of multiple myeloma [7,8,11].

Our review of recent articles revealed few case reports of plasmacytomas as initial presentations of multiple myeloma [9,11,12]. The locations of these reported masses include the clivus with extension towards the jugular foramen and the mandible [9], the sphenoid sinus with extension from the clivus [9], the skull base [12], and intracerebrally [12]. Despite identifying such a mass as plasmacytoma, additional tests are required to distinguish between a solitary plasmacytoma of the bone, an extramedullary plasmacytoma or the systemic disease multiple myeloma. Patients with solitary plasmacytoma of the bone are more likely to progress to multiple myeloma than those ith extramedullary plasmacytoma, but both conditions have a better overall prognosis than the systemic disease [9,12,13].

Our patient received radiation therapy during his hospital stay and was discharged to a skilled nursing facility to initiate chemotherapy. He and his family returned to their native Peru within two months of his discharge from the hospital.

## Conclusion

Failure to recognize the presentation of multiple myeloma leads to delays and even errors in diagnosis and treatment. When aspects of our patient's initial MRI did not correlate with the diagnosis with the greatest pretest probability (disc herniation), we were prompted to pursue follow-up studies and arrive at a correct, although surprising, conclusion. We do not suggest that a spinal mass resulting from multiple myeloma be kept at the forefront of the differential diagnosis of spinal cord compression. Rather, we present this case as an example of avoiding the anchoring heuristic by misdiagnosing lumbar disc protrusion [14].

## Competing interests

The authors declare that they have no competing interests.

## Authors' contributions

CC analyzed and interpreted our patient data regarding spinal cord compression and myeloma. KM was a major contributor in searching the current literature and writing the manuscript. Both authors read and approved the final manuscript.

## Consent

Written informed consent was obtained from our patient's next of kin for publication of this case report. A copy of the written consent is available for review by the Editor-in-Chief of this journal.
